# Developmental arcs of plasticity in whole movement repertoires of a clonal fish

**DOI:** 10.1016/j.isci.2025.113189

**Published:** 2025-07-23

**Authors:** Sean M. Ehlman, Ulrike Scherer, David Bierbach, Luka Stärk, Marvin Beese, Max Wolf

**Affiliations:** 1 Leibniz Institute of Freshwater Ecology and Inland Fisheries, 12587 Berlin, Germany; 2 SCIoI Exzellenzcluster, 10587 Berlin, Germany; 3 Humboldt Universität zu Berlin, 10115 Berlin, Germany

**Keywords:** Zoology, Neuroscience, Developmental neuroscience

## Abstract

Developmental plasticity at the behavioral repertoire level allows animals to incrementally adjust their behavioral phenotypes to match their environments through ontogeny. Quantifying this plasticity in sufficient resolution across substantial periods of development, however, has been challenging. Here, we use high-resolution tracking to monitor 45 genetically identical Amazon mollies (*Poecilia formosa)* reared in near-identical environments over their first four weeks of life. We analyze behavior at 0.2-s resolution to assess plasticity across entire behavioral repertoires. Testing a key prediction from Bayesian models—that plasticity should decline in stable environments—we measure plasticity using both individual behavioral metrics and a bespoke “behavioral entropy” approach in a multi-dimensional phenotype space. Surprisingly, and despite closely conforming to model assumptions, we find a consistent initial two-week *increase* in movement plasticity before a decline. These results challenge expectations about how plasticity unfolds early in life and highlight the importance of continuous behavioral tracking for evaluating developmental theories.

## Highlights

• A bespoke method quantifies multi-dimensional behavioral plasticity as behavioral entropy
• Clonal fish showed substantial arcs of plasticity through development
• This plasticity first increased for two weeks, then decreased
• Demonstrates the value of long-term tracking for testing developmental theory

## Graphical abstract

**Figure:**
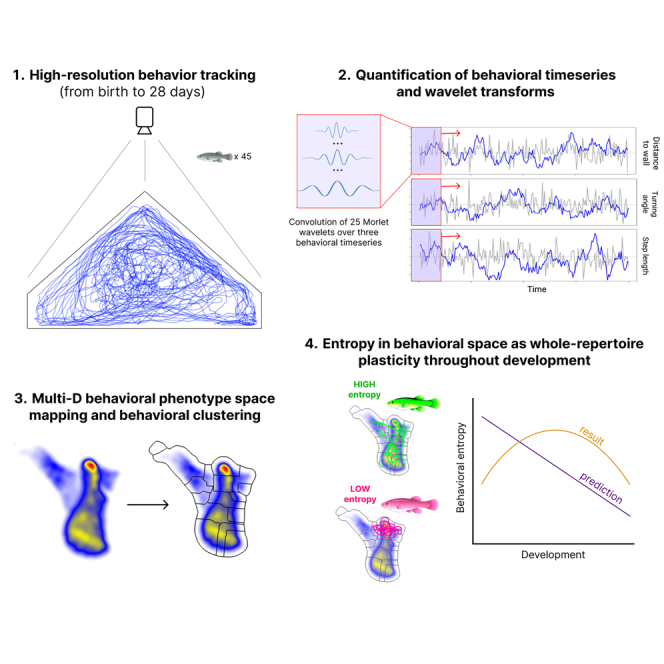

## Introduction

Exploration is a key aspect of early life for most animals, providing them with critical information about themselves and their environments. While new information encountered during exploration spurs phenotypic adjustments throughout life, the highest degree of plasticity (or behavioral flexibility) is often expected to occur early in life.^1^^,^^2^ This early life developmental plasticity may thus potentiate greater phenotypic integration, specialization, and/or competitive advantages compared to conspecifics that delay development.^3^^,^^4^^,^^5^^,^^6^ As development progresses, plasticity’s benefits (e.g., effective exploration of an environment, behavioral pattern, etc.) may be rebalanced against its costs (e.g., increased error rate, opportunity costs, etc.): in moderately stable and predictable environments, exploring environmental cues may not provide much new information^1^^,^^7^; aging animals may not have enough time left to capitalize on major plastic adjustments^8^; and while plasticity may offer efficient ways to explore an environment or a set of behaviors, this exploring may preclude exploiting already acquired information or further developing existing behavioral patterns.^9^^,^^10^ This rebalancing of the costs and benefits of plasticity across development governs individuals’ trajectories of plasticity across development and is a fundamental tenet of a large and influential theoretical literature on developmental plasticity^1^^,^^4^^,^^7^^,^^11^^,^^12^^,^^13^; in sharp contrast, studies that empirically document the arc of plasticity for any particular behavior—let alone multi-dimensional behavioral repertoires—throughout ontogeny have been exceedingly few (due in part to the time- and data-intensive nature of measuring behavior continually through long periods of development).

Our study builds on novel technological approaches to measuring behavior represented by automated tracking in high spatiotemporal resolution, allowing for near-continuous, long-term behavioral observation and the quantification of multi-dimensional movement repertoires.^14^^,^^15^^,^^16^^,^^17^^,^^18^^,^^19^^,^^20^ These advances present a path forward toward quantifying continuous plastic changes through development^21^ and may help to address a relative dearth of development-length behavioral datasets needed to address theory in this area.^22^ This study also offers a route to empirically test a basic and robust theoretical prediction that, in stable environments, given the potential benefits of exploring environments or behaviors early, plasticity (i.e., behavioral variability in response to salient cues from the environment) should monotonically decrease as individuals age.^7^^,^^13^ This prediction relies on the assumption that as naive animals repeatedly sample their environment and integrate new information, they improve their “estimate” of the environment (i.e., reduce uncertainty), leading to a decrease in sensitivity to new cues. While some empirical tests exist that test these basic predictions,^23^^,^^24^^,^^25^^,^^26^ few take a long-term developmental approach and measure behavior either continuously enough or at a resolution high enough to capture the trajectory of plasticity through ontogeny.

In this study, we address this gap. Tracking genetically identical fish at 0.2-s resolution from birth until four weeks of life (i.e., ∼1/3 of their total development to sexual maturity) in stable, highly standardized conditions, we develop a bespoke measure for mapping the continuous developmental arcs of behavioral plasticity. Whereas behavioral plasticity is typically measured in single behavioral dimensions (e.g., activity, exploration, etc.), we here quantify behavioral plasticity using a whole-repertoire approach that measures within-individual behavioral diversity as the degree of Shannon entropy in a high-dimensional behavioral hypervolume (i.e., “behavioral entropy”) during a prolonged period of development in which we assume individuals continuously “sample” their environment and adjust their behavioral repertoires accordingly. With this approach, we robustly find that individuals exhibit an inverted U-shape pattern of behavioral entropy through ontogeny in a stable environment, indicating an initial increase in movement repertoire plasticity until an average of ∼17 days after birth, followed by decreasing plasticity.

## Results

### Behavioral plasticity in individual behavioral metrics through ontogeny

We filmed 45 genetically identical Amazon mollies (*Poecilia formosa*) reared individually in near-identical environments^27^ from above, 8 hours a day from their first to 28^th^ day of life at five frames per second, yielding a timeseries of approximately 180 million x-y coordinate datapoints of fish in space. From these coordinates, we obtained three timeseries of the most basic behavioral metrics for which the original 0.2-s temporal resolution could be maintained: (1) an instantaneous measure of a fish’s activity, for which we used the Euclidean distance (“step length”) between two consecutive x-y coordinate points, (2) a measure of a fish’s bearing relative to their movement vector in the preceding timepoint (i.e., their “turning angle”), and (3) a measure of a fish’s position in space relative to a salient aspect of their environment, for which we chose the distance to the nearest tank wall.

**Figure 1:**
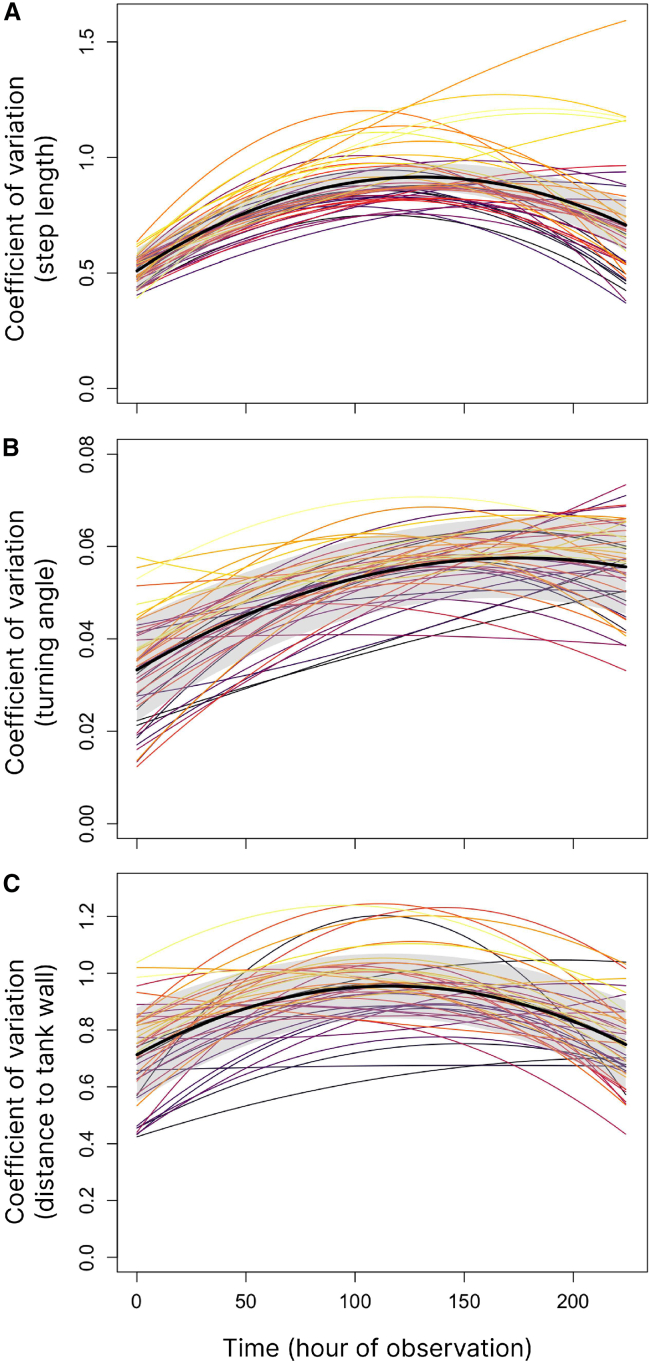
The relationship between developmental time and a measure of behavioral flexibility—the coefficient of variation (CoV)—for three basic measures of behavior (A) a measure of activity (here, “step length”), (B) a measure of a fish’s bearing (here, “turning angle”), and (C) a measure of relative spatial position (here, “distance to the tank wall”). A 95% confidence interval around the quadratic regression is shown in gray. Colored lines represent individual developmental trajectories (*n* = 45), the coefficients of which were obtained from a generalized linear mixed model with varying intercepts, linear, and quadratic coefficients by individual ID.

To quantify an individual’s behavioral plasticity in a particular trait through development, we calculated the coefficient of variation (CoV) for a given trait of a given individual by dividing their trait’s standard deviation by its mean for a given interval of time. Since the means and variances of all three basic behavioral metrics were positively correlated with each other, calculating the CoV for each trait isolated the effect of a trait’s variance from the correlated effect of its mean; this measure, when used to compare changes within a particular trait through time or contexts (rather than among traits), can be used as a measure of phenotypic flexibility or plasticity.^28^ In order to map the change in CoVs of each of the three basic behavioral metrics over the course of the first 28 days of individuals’ lives, CoVs were calculated over hour-long intervals throughout development for each individual, such that each individual had 8 h ∗ 28 days = 224 CoV measures for each of the behavioral metrics spanning the first 28 days of life. Each of an individual’s 224 CoV measures for each metric was thus ultimately based on 18k raw data points (each hour contained 18k raw data points). This yielded a timeseries of behavioral plasticity in each metric spanning the first 28 days of development. Selecting between linear and quadratic mixed models (Tables S1–S3) using their Akaike and Bayesian Information Criteria revealed that, in all three cases (step length, turning angle, and distance to the tank wall CoVs), quadratic models with inverted U-shapes and a peak of CoV between 110 and 180 h (between day ∼14–22) of observation (depending on the metric) had the highest support (Table S4). This indicated that behavioral plasticity in the three most basic behavioral metrics with time resolution that matches raw x-y coordinate measurements (5 Hz) exhibited an initial increase in behavioral plasticity lasting approximately 2.5 weeks, followed by a subsequent decrease (Figure 1).

### Behavioral plasticity as entropy in multi-dimensional behavioral phenotype space

**Figure 2:**
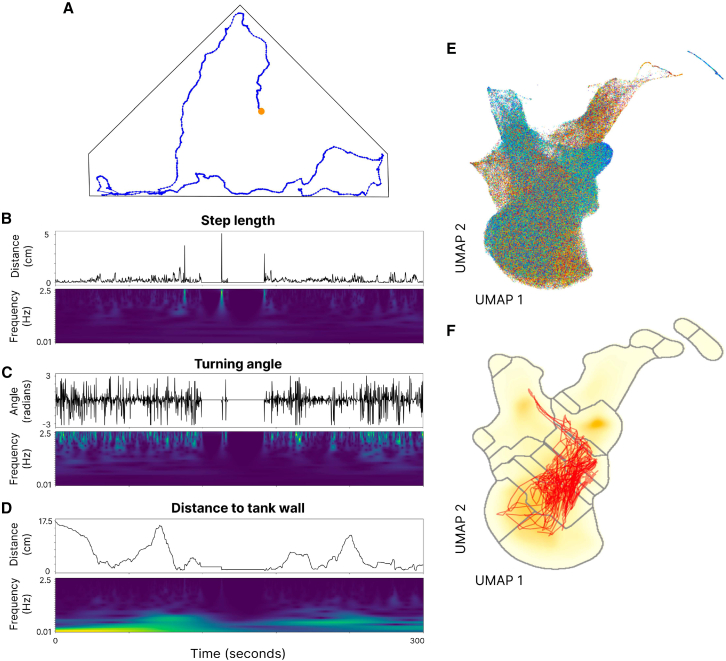
A schematic demonstrating the process used to convert an individual’s movement pattern in 2D physical space into a timeseries of “movement” through behavioral phenotype space (A) Here, a 300-s period of an individual’s movement in physical tank space (i.e., triangular shape) is recorded at 0.2-s resolution and plotted as a series of x-y coordinate points. The beginning of the movement sequence is indicated by an orange dot. Step length (B), turning angle (C), and distance to the nearest tank wall (D) are then directly calculated from the raw x-y coordinate data, producing a timeseries of these three metrics (upper graphs in B–D). Wavelet transforms are then applied to these (normalized) timeseries, with 25 distinct wavelets of frequencies ranging from 0.01 to 2.5 Hz, yielding a 75-dimensional feature space (a “behavioral phenotype space”). Shown in the lower graphs of (B–D) are spectrograms showing the amplitudes of the 25 Morlet wavelets on each timeseries (minimum amplitudes in dark blue for all spectrograms are 0 s; maximum amplitudes are in bright yellow and correspond to 0.65, 0.40, and 0.16 s for B, C, and D, respectively). (E) Data for all time points and all individuals represent a total of ∼180 million measured behavioral datapoints, each represented in the 75-dimensional behavioral phenotype space. Here, the behavioral phenotype space is represented in a dimensionally reduced UMAP embedding where different colors represent the 45 distinct individuals. This UMAP projection is downsampled 500x, and each point has 50% transparency. (F) Watershed clustering on the inverse of data density in the UMAP embedding serves as a method to grid behavioral space into *k* (here, 20) grid cells, over which behavioral entropy is calculated. The red trajectory showing “movement” in behavioral phenotype space for a single individual corresponds to the 300-s timeseries depicted in (A–D).

Our second approach to quantifying trajectories of plasticity across development sought to leverage the high dimensionality in our dataset to represent behavioral plasticity as the diversity of “movement” in multi-dimensional behavioral phenotype space, integrating across the basic behavioral metrics and the multiple scales of temporal autocorrelation potentially present in their timeseries (Figure 2). While the developmental arcs of behavioral plasticity represented by the relationships among developmental time and the CoVs of each of the three basic behavioral metrics provided an important validation step and foundation for further analyses, the multi-dimensional repertoire approach provided some distinct additional benefits: (i) including all metrics in a single measure of repertoire-wide plasticity allowed for the proportional representation of each metric in their contribution to the overall degree of total plasticity, (ii) while all metrics were represented at 5Hz resolution, plasticity in movement repertoires likely exists across a range of timescales, some much longer; the multi-dimensional repertoire approach thus involved wavelet transforms applied to the timeseries of basic behavioral metrics,^29^ creating additional feature dimensions that captured the degree of temporal autocorrelation in behavior (which may also represent important axes of behavioral variation, themselves^30^) across longer timescales in the basic behavior metrics (Figures 2B–2D; see STAR Methods).

After wavelet transforms were performed, data from all individuals and all timepoints throughout development represented a 75-dimensional behavioral phenotype space (Figure 2E). In order to quantify behavioral plasticity in this phenotype space over time, we calculated a measure of the spread of behaviors exhibited by any particular individual as their Shannon “behavioral entropy.” Derived from information theory, this same basic entropy measure^31^ is commonly used in biology to calculate biodiversity across a species assemblage^32^ or heterogeneity of physical space use in a landscape,^33^^,^^34^ and has also been applied to measure behavioral variability among a range of pre-defined behaviors^35^ and molecular diversity across genomes^36^; here we extend the application of entropy as a measure of an individual’s behavioral plasticity when entropy is calculated across a multi-dimensional, cluster-delineated behavioral phenotype space as a continuous function of time. Since Shannon entropy calculations in space generally require “space” to be discretized as a grid, gridding a 75-dimensional behavioral phenotype space was performed using unsupervised behavioral clustering via UMAP (Uniform Manifold Approximation and Projection^37^) embedding, Gaussian smoothing of data density in embedding space, and a watershed transform on the inverse of that density space^38^ (Figure 2F). Entropy was calculated over hour-long intervals through development for each individual, as with CoV measures, yielding 224 measures of entropy spanning the first 28 days of life for each individual.

**Figure 3:**
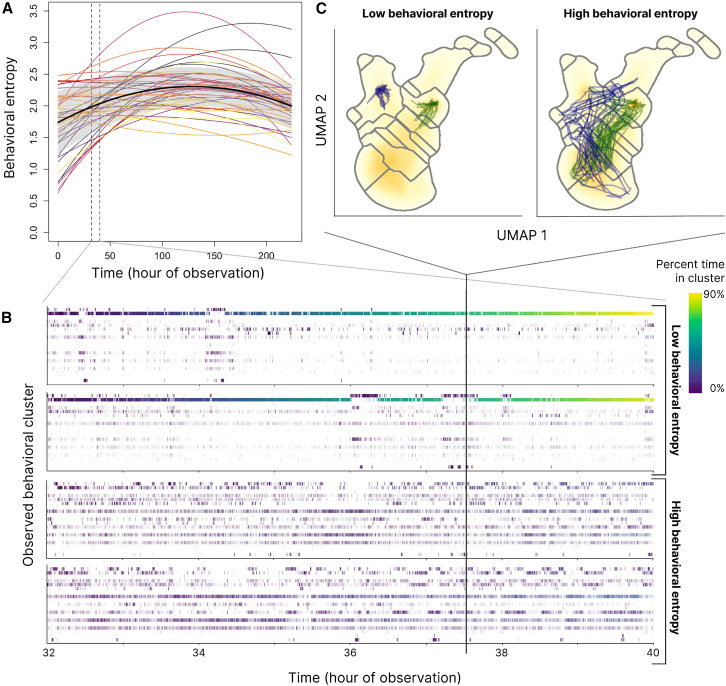
The developmental arcs of plasticity across the first 28 days of life for individuals, as represented by Shannon entropy calculated across a behavioral landscape (A) Average behavioral entropy in the population initially increases, peaking around observation hour 135 (∼17 days after birth), before then decreasing. Colored lines represent individual developmental trajectories (*n* = 45), the coefficients of which were obtained from a generalized linear mixed model with varying intercepts, linear, and quadratic coefficients by individual ID. A 95% confidence interval around this quadratic regression is shown in gray. (B) Representative day-length ethograms (from 8 continuous observation hours of four different individuals on day 5) in which cluster occupancy (cluster 1–20) is indicated through time at 0.2-s resolution by small vertical ticks colored by the cumulative time spent in that particular cluster during the observation interval shown. The top two ethograms demonstrate a pattern of low behavioral entropy over this interval, while the bottom two ethograms represent a pattern of high entropy. (C) Three-minute trajectories (corresponding in time to the vertical solid line shown in (B)) through behavioral phenotype space represented as a non-linear embedding via UMAP. Watershed cluster boundaries are shown; the two low entropy individuals shown in (B) are represented in the left UMAP, while the two high entropy individuals shown in (B) are in the right UMAP.

As with CoV measures, the developmental time course of entropy was best described as an inverted U-shape (Tables S3 and S4): Figure 3A shows developmental arcs of behavioral entropy in which behavioral plasticity initially increases, peaking around hour 135 (∼17 days) before then decreasing. Individual fish (colored lines) vary in their mean entropy through development, and it is instructive to contrast behavioral patterns of relatively low- and high-entropy individuals. Figure 3B shows, for example, that individuals with low behavioral entropy exhibit patterns of reduced “movement” in behavioral space, with some low entropy individuals spending up to 90% of their time in any given hour in a single behavioral cluster (Figure 3B). High entropy individuals, in contrast, exhibit a more uniform distribution of cluster occupancy, spending approximately equal amounts of time in a larger number of behavioral clusters during each hour. This can be represented as trajectories through behavioral phenotype space (Figure 3C): even though any two low entropy individuals might exhibit quite different individual behaviors (i.e., be segregated in behavioral phenotype space), they are united in their limited movement through total behavioral space, in contrast to high entropy individuals who use a much greater proportion of behavioral phenotype space. The patterns of greater “movement” in behavioral space (and thus higher behavioral entropy) correspond to a greater diversity of movement patterns in physical space when simultaneous fish movements in physical space and behavioral (UMAP) space are visualized side-by-side (Video S1). These results are further validated by and extend the standard but more limited approach to quantifying patterns of single-behavioral-dimension plasticity (CoVs) in the basic behavioral metrics shown earlier (Figure 1).

Video S1. A 5-min movie depicting low- and high-entropy individuals in both physical tank space and behavioral phenotype space A 5-min movie in which three low entropy individuals (A) and three high entropy individuals (B) are shown exhibiting a continuous track of behavior over a 30-min real-time sequence (6x speed) in both physical tank space (triangular shapes on the left of each panel) and clustered behavioral phenotype (UMAP) space. This movie is related to and referenced in the “behavioral plasticity as entropy in multi-dimensional behavioral phenotype space” section in the main text.

## Discussion

We mapped the developmental arcs of behavioral plasticity in entire movement repertoires of 45 genetically identical fish reared in near-identical environments for the first 28 days of their lives at 0.2 s resolution. Employing a novel measure of entropy in behavioral repertoire space, we then addressed a key prediction from Bayesian models of development that, in stable environments, behavioral plasticity should gradually decrease as (initially) naive individuals age.^7^^,^^13^^,^^39^ In our experimental setup, fish experienced constant and benign conditions from their day of birth through the duration of the experiment; despite this, matching as best as possible the assumptions of models that predict decreasing plasticity, our results indicate a ∼two-week initial increase in plasticity in movement behaviors before plasticity subsequently decreased.

The deviation of our empirical results from those expected by theory might be explained by discontinuities between informational priors (e.g., those set evolutionarily or via parental effects) and individually sampled information.^40^^,^^41^^,^^42^^,^^43^^,^^44^^,^^45^ It is possible, for example, that newborn individuals—small prey fish vulnerable to high levels of predation in natural streams^46^—start life with a relatively conservative “prior” about the level of risk (i.e., are not, in fact, “naive”), and thus show an initial increase in plasticity early in life as they sample to rebalance their erroneous estimate of risk (e.g., habituate) in now-predator-free, benign lab conditions. Alternatively, while predicted developmental trajectories of plasticity in Bayesian models of development are driven primarily by informational constraints (e.g., trade-offs involved in “exploring” new information or “exploiting” already sampled information), there may be other developmental constraints, such as motor or cognitive constraints,^47^^,^^48^ that otherwise restrict the behavioral repertoire range of newly born mollies, delaying periods of maximal plasticity until later in development.^49^ Whatever the underlying cause explaining the deviation of our empirical results from those predicted by basic Bayesian theory, we emphasize the utility in applying recent advancements in high-resolution behavioral tracking to test and refine open questions in the theory of behavioral development.

Furthermore, while plasticity is often measured in response to specifically defined or experimentally induced stimuli,^50^^,^^51^ we here infer plasticity by quantifying within-individual behavioral variability (aka behavioral flexibility) over a prolonged period of time (here, an hour or a day) in which individuals can continuously “sample” their environment and adjust their behaviors in response. Changes in plasticity through development are thus inferred by comparing the range of within-individual behavioral diversity in whole repertoires across defined intervals of time throughout development. In any case, the quantification of the whole repertoire behavioral entropy through development permits us to test the general intuition derived from the most basic Bayesian models of development that, in stable environments, individuals should developmentally hone their set of behaviors, reflecting a more assured picture of their world obtained through repeated sampling. This process is thus predicted to result in an initially diverse set of behaviors that is progressively whittled into a more refined repertoire suited to their particular environment. Importantly, this basic intuition functions equally well as a starting point in understanding a wide range of behavioral and cognitive changes throughout development, from plastic changes in reproductive strategies of guppies,^23^ developmental changes in cognitive flexibility and exploration of human children^24^^,^^25^ – and here, to changes in whole repertoire behavioral entropy across ontogeny of Amazon mollies.

Our results add to and significantly extend a nascent but growing set of studies using high-throughput behavior tracking and multi-dimensional behavioral representations to document behavioral repertoire changes throughout time^52^^,^^53^^,^^54^^,^^55^^,^^56^^,^^57^^,^^58^; in particular, our results demonstrate the value of applying emerging technologies to the continuous measurement of behavioral plasticity during ontogeny. This is particularly important given the long-held consensus that more continuous measurements of behavior and its plasticity through development are critical in advancing our understanding of the ecological and evolutionary factors shaping behavior and its development.^22^^,^^59^ Such “big behavioral data” approaches to quantifying and analyzing behavior and its plasticity could be productively applied to a range of important questions in behavioral development, including: the development of individual differences in plasticity,^60^^,^^61^^,^^62^^,^^63^ developmental constraints on patterns of behavioral variation,^64^ and the role of developmental plasticity in mediating behavioral responses to environmental change.^65^^,^^66^^,^^67^^,^^68^ Given the crucial role of developmental plasticity in both translating ecological cues into phenotypic effects and producing the range of phenotypes upon which selection acts,^69^^,^^70^^,^^71^ new tools for fully mapping the continuous arcs of such developmental plasticity in behavior are likely to significantly contribute to our overall understanding of the ecological and evolutionary causes and consequences of behavioral development.

### Limitations of the study

While we have documented the developmental time course of behavioral variation for individuals reared in stable, highly standardized environments, future studies would likely benefit from an explicit consideration of the effects of environmental complexity or predictability on the expression of flexibility or plasticity in behavioral repertoires. Environmental predictability, for example, is known to affect plasticity as well as cognitive flexibility and learning.^6^^,^^72^^,^^73^ Because environmental conditions may affect the development of behavioral plasticity in any particular trait, and plasticities among many traits may be correlated,^74^ such effects could have large effects at the level of the whole repertoire. Additionally, future studies in which informational priors of individuals are manipulated early in development (e.g., through the manipulation of parental experiences) and in which even longer periods of life are measured may allow for more complete tests of the range of predictions from Bayesian models of development, such as under which set of conditions plasticity is predicted to be prolonged, non-monotonic, or even increasing throughout ontogeny.^45^ Although we measured fish for a full third of their development, longer periods of measurement up to the time of sexual maturity may provide additional insight on, for example, the stability of the patterns of plasticity we document here.

Furthermore, while predictions from many of the Bayesian models used to predict early life plasticity are equally applicable to animals in later life (with the usual distinction being that the strengths of their priors likely differ based on differences in experience), explicit comparisons among individuals at different life stages would provide exciting additional avenues to assess the degree to which the patterns documented here are particular to early life stages in the Amazon molly. Lastly, while predictions on the developmental course of behavioral flexibility can be derived from Bayesian models of development, a range of alternative drivers or constraints of plasticity through development should be explored. Indeed, as mentioned earlier, there may also be motor, sensory, or other constraints (either in addition to or alternative to the informational constraints posited by Bayesian models of development) that may influence the range of behaviors available to individuals at various development timepoints. In addition to future experiments that manipulate the informational priors, for example, a range of additional measures that quantify alternative constraints may provide new insight into the diversity of drivers of behavioral plasticity through development. Thus, we call for a range of alternative experimental controls and measures that will allow for more clarity on the underlying processes driving the observed patterns of plasticity through development. We also stress the utility of whole-repertoire measures such as that presented here in quantifying arcs of plasticity in such contexts.

## Resource availability

### Lead contact

Information and requests for resources should be directed to and will be fulfilled by the Lead Contact, Sean M. Ehlman (sehlman@mailbox.sc.edu).

### Materials availability

The study did not generate new unique reagents.

### Data and code availability

• Data: The full dataset of fish behavioral tracks associated with this publication is publicly available in a Dryad data repository (https://doi.org/10.5061/dryad.x69p8cztw) and is also listed in the key resources table. The full dataset is a timeseries, measured at 5Hz, of x-y coordinate points, representing fish movements from the first day of life to day 28. Also included are the calculated metrics of step length, turning angle, and distance to the tank wall.
• Code: All associated code needed to reproduce the results in this publication has been deposited in a publicly accessible repository (https://github.com/smehlman/behavioral_entropy).
• Additional Information: Any additional information required to reanalyze the data reported in this article is available from the lead contact upon request.

## Acknowledgments

We gratefully acknowledge funding from the 10.13039/501100001659Deutsche Forschungsgemeinschaft (DFG) under Germany's Excellence Strategy EXC 2002/1, “Science of Intelligence” (project number #390523135) and from a 10.13039/501100001659DFG “Eigene Stelle” grant to SME (#536703956). Members of the Wolf and Krause labs at the Leibniz Institute of Freshwater Ecology and Inland Fisheries, Humboldt University, and SCIoI Exzellenzcluster provided invaluable help with animal husbandry, experimental design and logistics, as well as coding and data management: particular thanks are extended to F. Francisco, J. Krause, C. Schutz, D. Strasiewsky, J. Piotrowski, P. Schladitz, F. Bernstein, S. Wersing, and O. O’Connor. We are grateful also to N. Walasek for helpful discussions and comments on the article. The article also benefited from helpful comments by three reviewers and the editor.

## Author contributions

SME, US, DB, and MW conceived of the project idea and designed the experiment. MW secured funding for the experiment. DB designed the housing tanks and tracking system. US conducted the experiment. SME led analyses. SME, LS, and MB conducted analyses. SME wrote the first draft of the article. SME, US, DB, LS, MB, and MW edited article drafts.

## Declaration of interests

We declare no competing interests.

## STAR★Methods

### Key resources table

**Table:** 

REAGENT or RESOURCE	SOURCE	IDENTIFIER
**Deposited data**		

Full dataset of fish behavioral tracks (x-y coordinate points recorded at 5 Hz) from day 1 to day 28 in lives of Amazon mollies	This paper	Dryad data repository: Dataset for ‘Developmental arcs of plasticity in whole movement repertoires of a clonal fish' https://doi.org/10.5061/dryad.x69p8cztw

**Experimental models: Organisms/strains**		

Amazon mollies (*Poecilia formosa*)	Humboldt Universität zu Berlin lab stock	N/A

**Software and algorithms**		

Code used for analysis of data	This paper	Github repository: https://github.com/smehlman/behavioral_entropy

### Experimental model and subject details

All Amazon mollies (*Poecilia formosa*) used in this study derived from a single isogenic stock kept at Humboldt Universität zu Berlin (Berlin, Germany). All animal care and experimental protocols complied with local and federal laws and guidelines and were approved by the appropriate governing body in Berlin, Germany, the Landesamt fur Gesundheit und Soziales (LaGeSo G-0224/20).

### Method details

#### Experimental design

Details on study species, animal housing and feeding protocols, and experimental design are described in^27^; we briefly describe them here. In this study, genetically identical gravid Amazon mollies (*Poecilia formosa*) were isolated from a single isogenic stock kept at Humboldt Universität zu Berlin (Berlin, Germany). The Amazon molly is a gynogenetic species of freshwater fish^75^^,^^76^—the first described species of clonal vertebrate^77^—with a diverse behavioral repertoire through development.^27^^,^^78^^,^^79^^,^^80^ Offspring from three of these isogenic mothers were used as experimental animals in behavioral observations. In addition to the original three mothers of experimental animals being genetically identical and arising from the same stock tank, we also accounted for individual mother ID in all statistical models (Figures S2 and S3; Tables S1–S11). In this way, we both minimized any maternal effects due to obvious differential experiences of mothers and ensured that all experimental animals were genetically identical. In total, three mothers provided 45 experimental fish, which were transferred on the day of their birth to large individual observation tanks (Figure S1). From the next day (their first full day of life) until an age of 28 days, fish were filmed from above using a Basler acA5472-5 gm camera fitted with a 16 mm lens (Basler Lens C11-1620-12M-P f16mm) at five frames per second continuously for eight hours per day. Given that mollies reach sexual maturity at approximately three months, behavioral observations covered a full third of development to maturity for these fish. Note that while overhead filming meant that the third (i.e., vertical) dimension of movement was not recorded, the water level in tanks was kept relatively shallow (at a depth of ∼7cm) so that the dominant dimensions of possible movement were largely confined to two dimensions. Fish were kept on a 12:12h light:dark cycle with an air temperature of approximately 24 ± 1°C and fed daily during a two hour period following the 8-hour filming period with a stationary ‘food patch’ consisting of Sera vipan baby fish food fixed in agar. Observation tanks were illuminated from below with four LEDs per tank (each LED was 100cm in length, 12V, color temperature = 5500 K, light output = ∼1570 lumen); tanks were manufactured from white polyethylene, which allowed for diffuse light from LEDs to illuminate the tank evenly. Illumination from below minimized glare on the water surface and allowed for ease of automated video tracking conducted using the software Biotracker.^81^ This generated data in the form of a timeseries of fish positions in x-y coordinate space with 0.2 second temporal resolution.

### Quantification and statistical analyses

#### Statistical models for basic behavioral measures

For each basic behavioral metric (i.e., step length, turning angle, and distance to the tank wall), we calculated a trait’s coefficient of variation (CoV) by dividing its standard deviation by its mean for a given interval of time at hour-length intervals (constituting 18k raw data points for each CoV datapoint). See Figure S2 and Tables S5–S8 for further analyses using CoVs calculated over day-length intervals; note that results were robust to the specific interval used to calculate individual CoV datapoints). Visual inspection of the course of plasticity in all three basic behavioral traits revealed inverted U-shapes rather than monotonic decreases in plasticity; this was confirmed by model comparison between linear and quadratic models in a mixed modeling framework with a main fixed effect of developmental time, controlling for mother ID, tank position within the experimental room (center or periphery within a flow-through system), and tank system (one of four flow-through systems) as additional fixed effects. Individual ID was treated as a random effect, with randomly varying intercepts and slopes in the case of linear models and randomly varying intercepts, linear, and quadratic coefficients in the case of quadratic models (see Tables S1–S4 for further details).

#### Multi-dimensional behavioral phenotype space

In order to create a multi-dimensional behavioral phenotype space, we first performed wavelet transforms on each of the three (normalized) basic behavioral metric timeseries following^29^^,^^82^: 25 wavelets of different frequencies (min. frequency: 0.01 Hz; max. frequency: 2.5 Hz) were convolved over the three basic behavioral measure timeseries yielding a total of 75 feature dimensions. The convolution of a wavelet over a behavioral timeseries yields an ‘activation’ at each point in the timeseries, which is a measure of the waveform’s ‘goodness of fit’ between a timeseries and that waveform centered at a particular point along that timeseries.^83^ The timeseries of a wavelet’s activations filtered over a behavioral timeseries thus becomes its own timeseries. By using a basic waveform with short-lived localized oscillations (here, the Morlet wavelet^84^), changing the frequency of that waveform, and comparing activations of these waveforms with different frequencies on a behavioral timeseries, variable patterns of temporal autocorrelation in the data can be captured as additional features in behavioral repertoire space. Thus, by wavelet transforming spatial positioning, activity, and turning angles, we can jointly represent behavioral phenotype space as encompassing variation in the data of where animals are, how and how quickly they move, as well as the varying degrees of temporal autocorrelation in these measures.

#### Behavioral entropy measure

After wavelet transforms were performed, data from all individuals and all timepoints through development represented a 75-dimensional behavioral phenotype space. In order to quantify ‘movement’ through this phenotype space over time, we calculated a measure of the spread of behaviors exhibited by any particular individual over all of behavioral space as their ‘behavioral entropy’: the greater behavioral diversity individuals exhibited across the total behavioral space, the greater their behavioral entropy and thus plasticity. In order to calculate entropy, behavioral space first needed to be partitioned, which was performed through the use of a non-linear dimension reduction step (Uniform Manifold Approximation and Projection; UMAP^37^) followed by Gaussian smoothing of data density in embedding space and a watershed transform on the inverse of that density space.^38^ This yielded a specified number of behavioral clusters; for the main analysis, we used 20 clusters (i.e., 20 ‘grid cells’ over which entropy was calculated), but results were robust to much lower cluster numbers (Figure S3; Tables S9–S11). Figure S3 and Tables S9–S11 also contain additional analyses with an alternative clustering algorithm (k-means performed over the full 75-dimension behavioral space), and results were again qualitatively unchanged.

Once behavioral clusters were identified, Shannon entropy over behavioral space, *H*(*X*), was calculated as:

$$H\left(X\right)=-\sum_{i}P\left(x_{i}\right)logP\left(x_{i}\right)$$

where *P*(*x*_*i*_) is the probability of being in any cluster, *i*, computed from the data as the proportion of time a fish ‘visited’ a particular cluster (i.e., exhibited a behavior within a behavioral cluster). In order to calculate the timeseries of entropies for each individual fish—and thus plasticity in their movement repertoire—over development, hourly entropy for each fish was calculated by binning behavioral timeseries into 1-hour intervals. Note that behavioral entropy was also calculated on a day-length interval (Figure S2; Tables S5–S8); results do not differ qualitatively across these interval scales. Lastly, in order to assess the specific shape of developmental arcs in behavioral plasticity (i.e., entropy), we again employed a model comparison approach between linear and quadratic models in a mixed modeling framework with a main fixed effect of developmental time, controlling for mother ID, tank position within the experimental room (center or periphery within a flow-through system), and tank system (one of four flow-through systems) as additional fixed effects. Individual ID was treated as a random effect, with randomly varying intercepts and slopes in the case of linear models and randomly varying intercepts, linear, and quadratic coefficients in the case of quadratic models (see Tables S1–S4, for further details). All linear mixed effects models (for both CoV measures and entropy) were conducted using the lme4 package^85^ in R version 4.1.1.^86^ All other analyses (e.g., wavelet transforms, UMAP embedding and watershed clustering) were performed in Python version 3.9.
