# Supplementary material for: Developmental arcs of plasticity in whole movement repertoires of a clonal fish
Source: iScience. 2025 Jul 23;28(9):113189. doi: 10.1016/j.isci.2025.113189 (PMC12397953; doi:10.1016/j.isci.2025.113189)
Supplement: Document S1. Figures S1–S3 and Tables S1–S11 [file mmc1.pdf]

iScience, Volume *28*

Supplemental information

Developmental arcs of plasticity in whole movement repertoires of a clonal fish

Sean M. Ehlman, Ulrike Scherer, David Bierbach, Luka Stärk, Marvin Beese, and Max Wolf

<u>Observation tank setup</u>

Figure S1. The layout of the experimental tanks in which fish were housed and filmed from above for the first 28 days of life. Each experimental unit (shown here) contained two experimental tanks, and experimental units were connected via flow-through water systems. Food patches were only present during the two-hour feeding period, outside of the eight-hour behavioral observation periods included in analyses. Reproduced with permission from [1]. This figure is related to the STAR Methods subsection, '*Experimental design*'.

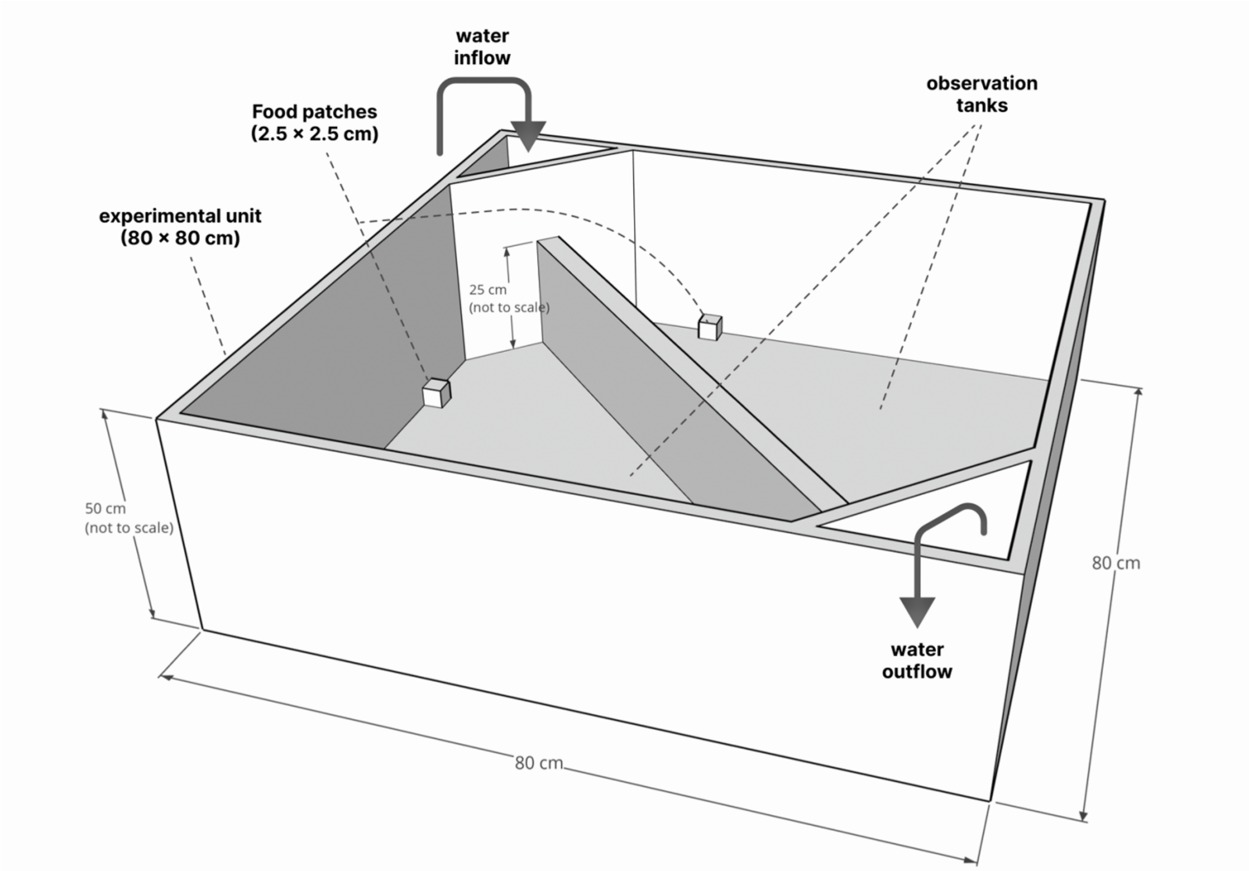

Model output and selection

Table S1. Output for the linear models of the coefficient of variation (CoV) for step length, turning angle, and distance to the tank wall regressed against time (in hours) as the continuous fixed predictor of interest. Mother ID, tank position, and tank system are included as categorical fixed predictors, and fish ID is included as a random factor with varying slopes and intercepts. P-values less than a 0.05 significance threshold are bolded. This table is related to the STAR Methods subsection '*Statistical models for basic behavioral measures*'.

	**Step length CoV (linear model)**			**Turning angle CoV (linear model)**			**Distance to tank wall CoV (linear model)**		
Predictors	*Estimates*	*CI*	*p*	*Estimates*	*CI*	*p*	*Estimates*	*CI*	*p*
(Intercept)	0.696	0.612 – 0.779	**<0.001**	0.040	0.034 – 0.045	**<0.001**	0.850	0.761 – 0.939	**<0.001**
timestep	0.085	0.046 – 0.123	**<0.001**	0.010	0.008 – 0.012	**<0.001**	0.014	-0.012 – 0.040	0.292
mother ID [2]	-0.013	-0.116 – 0.091	0.813	-0.004	-0.011 – 0.003	0.230	-0.125	-0.234 – -0.015	**0.025**
mother ID [3]	-0.051	-0.105 – 0.004	0.069	0.004	0.001 – 0.008	**0.014**	-0.007	-0.064 – 0.051	0.819
tank position [middle]	0.010	-0.053 – 0.074	0.755	0.001	-0.003 – 0.005	0.695	-0.007	-0.073 – 0.060	0.846
tank position [wall]	-0.004	-0.071 – 0.063	0.912	0.001	-0.004 – 0.005	0.807	0.005	-0.066 – 0.075	0.900
tank system [2]	0.010	-0.062 – 0.081	0.789	-0.001	-0.005 – 0.004	0.734	0.075	-0.000 – 0.150	0.051
tank system [3]	0.006	-0.070 – 0.081	0.883	0.001	-0.004 – 0.005	0.814	0.018	-0.062 – 0.097	0.665
tank system [4]	-0.029	-0.101 – 0.043	0.424	-0.003	-0.008 – 0.001	0.183	0.001	-0.075 – 0.076	0.989
**Random Effects**									
σ^2	0.17589			0.00009			0.05574		
τ_{00}	0.01916 $_{id}$			0.00014 $_{id}$			0.02714 $_{id}$		
τ_{11}	0.01550 $_{id.timestep}$			0.00004 $_{id.timestep}$			0.00734 $_{id.timestep}$		
ICC	0.07270			0.36940			0.18616		
N	45 $_{id}$			45 $_{id}$			45 $_{id}$		
Observations	9861			9861			9861		
Marginal R^2 / Conditional R^2	0.020 / 0.091			0.245 / 0.524			0.031 / 0.211		

Table S2. Output for the quadratic models of the coefficient of variation (CoV) for step length, turning angle, and distance to the tank wall regressed against time and time^2 (in hours) as the continuous fixed predictors of interest. Mother ID, tank position, and tank system are included as categorical fixed predictors, and fish ID is included as a random factor with varying intercepts, quadratic, and linear coefficients. P-values less than a 0.05 significance threshold are bolded. This table is related to the STAR Methods subsection '*Statistical models for basic behavioral measures*'.

	**Step length CoV (quadratic model)**			**Turning angle CoV (quadratic model)**			**Distance to tank wall CoV (quadratic model)**		
Predictors	*Estimates*	*CI*	*p*	*Estimates*	*CI*	*p*	*Estimates*	*CI*	*p*
(Intercept)	0.509	0.414 – 0.605	**<0.001**	0.033	0.022 – 0.044	**<0.001**	0.713	0.547 – 0.879	**<0.001**
timestep^2	-0.238	-0.276 – -0.200	**<0.001**	-0.008	-0.010 – -0.006	**<0.001**	-0.178	-0.215 – -0.141	**<0.001**
timestep	0.621	0.543 – 0.700	**<0.001**	0.028	0.023 – 0.032	**<0.001**	0.415	0.328 – 0.503	**<0.001**
mother ID [2]	-0.202	-0.332 – -0.072	**0.002**	-0.014	-0.029 – 0.001	0.072	-0.239	-0.467 – -0.011	**0.040**
mother ID [3]	-0.058	-0.125 – 0.009	0.087	0.003	-0.005 – 0.011	0.429	0.072	-0.048 – 0.192	0.238
tank position [middle]	0.007	-0.071 – 0.085	0.869	0.001	-0.008 – 0.010	0.819	0.003	-0.137 – 0.142	0.972
tank position [wall]	-0.011	-0.093 – 0.071	0.790	-0.003	-0.013 – 0.007	0.538	-0.092	-0.239 – 0.055	0.219
tank system [2]	0.040	-0.048 – 0.128	0.371	0.006	-0.005 – 0.016	0.267	0.060	-0.097 – 0.217	0.452
tank system [3]	-0.015	-0.108 – 0.078	0.757	0.001	-0.010 – 0.012	0.825	-0.063	-0.229 – 0.103	0.459
tank system [4]	-0.024	-0.112 – 0.065	0.601	-0.003	-0.014 – 0.008	0.578	0.002	-0.156 – 0.160	0.981
**Random Effects**									
σ^2	0.16549			0.00008			0.04964		
τ_{00}	0.00688 $_{id.2}$			0.00016 $_{id.2}$			0.03453 $_{id.2}$		
τ_{11}	0.04112 $_{id.timestep}$			0.00023 $_{id.timestep}$			0.08048 $_{id.timestep}$		
	0.01136 $_{id.I(timestep^2)}$			0.00004 $_{id.I(timestep^2)}$			0.01423 $_{id.I(timestep^2)}$		
ICC	0.29347			0.82355			0.73049		
N	45 $_{id}$			45 $_{id}$			45 $_{id}$		
Observations	9861			9861			9861		
Marginal R^2 / Conditional R^2	0.056 / 0.333			0.146 / 0.849			0.072 / 0.750		

Table S3. Output for linear and quadratic models of behavioral entropy (calculated on a grid of 20 clusters obtained via UMAP dimension reduction and watershed segmentation) regressed against time (in hours) as the continuous fixed predictor of interest. The quadratic model contains an additional term for $time^2$ as a continuous fixed predictor. Mother ID, tank position, and tank system are included as categorical fixed predictors, and fish ID is included as a random factor with varying intercepts and linear coefficients (as well as varying quadratic coefficients in the case of the quadratic model). P-values less than a 0.05 significance threshold are bolded. This table is related to the STAR Methods subsection '*Behavioral entropy measure*'.

	**Behavioral entropy (linear model)**			**Behavioral entropy (quadratic model)**		
Predictors	*Estimates*	*CI*	*p*	*Estimates*	*CI*	*p*
(Intercept)	1.68	1.54 – 1.82	**<0.001**	1.74	1.25 – 2.23	**<0.001**
timestep	0.11	0.03 – 0.19	**0.007**	0.88	0.63 – 1.14	**<0.001**
mother ID [2]	-0.13	-0.23 – -0.02	**0.017**	-1.15	-1.82 – -0.47	**0.001**
mother ID [3]	-0.08	-0.13 – -0.02	**0.005**	-0.23	-0.59 – 0.12	0.201
tank position [middle]	0.02	-0.05 – 0.08	0.593	-0.12	-0.53 – 0.29	0.570
tank position [wall]	0.03	-0.03 – 0.10	0.306	-0.33	-0.76 – 0.11	0.143
tank system [2]	0.06	-0.01 – 0.13	0.087	0.29	-0.18 – 0.75	0.222
tank system [3]	0.01	-0.06 – 0.09	0.754	-0.19	-0.68 – 0.31	0.460
tank system [4]	-0.03	-0.10 – 0.05	0.491	-0.19	-0.66 – 0.28	0.434
timestep^2				-0.34	-0.43 – -0.25	**<0.001**
**Random Effects**						
σ^2	0.12			0.10		
τ_{00}	0.17 $_{id}$			0.32 $_{id.2}$		
τ_{11}	0.08 $_{id.timestep}$			0.74 $_{id.timestep}$		
				0.08 $_{id.I(timestep^2)}$		
ICC	0.27			0.93		
N	45 $_{id}$			45 $_{id}$		
Observations	9861			9861		
Marginal R^2 / Conditional R^2	0.048 / 0.306			0.097 / 0.934		

Table S4. Table of Akaike Information Criteria (AIC) and Bayesian Information Criteria (BIC) used to compare linear and quadratic models (see Tables S1, S2, and S3) for each response. This table is related to the STAR Methods subsections '*Statistical models for basic behavioral measures*' and '*Behavioral entropy measure*'.

**Model**	**ΔAIC**	**ΔBIC**
Step length CoV quadratic	0.00	0.00
Step length CoV linear	468.68	461.48
Turning angle CoV quadratic	0.00	0.00
Turning angle CoV linear	1250.12	1242.92
Distance to tank wall CoV quadratic	0.00	0.00
Distance to tank wall CoV linear	865.40	858.20
Behavioral entropy quadratic	0.00	0.00
Behavioral entropy linear	1801.03	1793.83

Developmental arcs of plasticity with day-length time intervals

Table S5. Output for the linear models of the coefficient of variation (CoV) for step length, turning angle, and distance to the tank wall regressed against time (in days) as the continuous fixed predictor of interest. Mother ID, tank position, and tank system are included as categorical fixed predictors, and fish ID is included as a random factor with varying slopes and intercepts. P-values less than a 0.05 significance threshold are bolded. This table is related to the STAR Methods subsection '*Statistical models for basic behavioral measures*'.

	**Step length CoV (linear model – day interval)**			**Turning angle CoV (linear model – day interval)**			**Distance to tank wall CoV (linear model – day interval)**		
Predictors	*Estimates*	*CI*	*p*	*Estimates*	*CI*	*p*	*Estimates*	*CI*	*p*
(Intercept)	0.762	0.673 – 0.851	**<0.001**	0.041	0.035 – 0.046	**<0.001**	0.890	0.796 – 0.983	**<0.001**
timestep	0.556	0.218 – 0.894	**0.001**	0.077	0.061 – 0.092	**<0.001**	0.139	-0.085 – 0.363	0.223
mother ID [2]	-0.027	-0.140 – 0.085	0.637	-0.004	-0.011 – 0.003	0.229	-0.131	-0.246 – -0.016	**0.025**
mother ID [3]	-0.013	-0.072 – 0.045	0.656	0.004	0.001 – 0.008	**0.018**	-0.013	-0.073 – 0.048	0.684
tank position [middle]	0.003	-0.066 – 0.071	0.934	0.001	-0.003 – 0.005	0.769	-0.010	-0.080 – 0.060	0.785
tank position [wall]	-0.004	-0.076 – 0.068	0.911	0.000	-0.004 – 0.005	0.836	-0.008	-0.082 – 0.066	0.837
tank system [2]	0.013	-0.064 – 0.091	0.731	-0.001	-0.005 – 0.004	0.707	0.062	-0.017 – 0.141	0.122
tank system [3]	0.009	-0.073 – 0.091	0.831	0.001	-0.004 – 0.005	0.784	-0.004	-0.087 – 0.080	0.929
tank system [4]	-0.027	-0.105 – 0.050	0.488	-0.003	-0.008 – 0.001	0.177	0.001	-0.079 – 0.081	0.978
**Random Effects**									
σ^2	0.04918			0.00006			0.02545		
τ_{00}	0.01522 $_{id}$			0.00013 $_{id}$			0.02608 $_{id}$		
τ_{11}	1.05240 $_{id.timestep}$			0.00252 $_{id.timestep}$			0.43676 $_{id.timestep}$		
ICC	0.24630			0.47398			0.32466		
N	45 $_{id}$			45 $_{id}$			45 $_{id}$		
Observations	1239			1239			1239		
Marginal R^2 / Conditional R^2	0.033 / 0.272			0.295 / 0.629			0.054 / 0.361		

Table S6. Output for the quadratic models of the coefficient of variation (CoV) for step length, turning angle, and distance to the tank wall regressed against time and time^2 (in days) as the continuous fixed predictors of interest. Mother ID, tank position, and tank system are included as categorical fixed predictors, and fish ID is included as a random factor with varying intercepts, quadratic, and linear coefficients. P-values less than a 0.05 significance threshold are bolded. This table is related to the STAR Methods subsection '*Statistical models for basic behavioral measures*'.

	**Step length CoV (quadratic model – day interval)**			**Turning angle CoV (quadratic model – day interval)**			**Distance to tank wall CoV (quadratic model – day interval)**		
Predictors	*Estimates*	*CI*	*p*	*Estimates*	*CI*	*p*	*Estimates*	*CI*	*p*
(Intercept)	0.521	0.419 – 0.623	**<0.001**	0.035	0.026 – 0.045	**<0.001**	0.686	0.564 – 0.808	**<0.001**
timestep^2	-14.09	-16.250 – -11.940	**<0.001**	-0.507	-0.585 – -0.428	**<0.001**	-11.44	-12.95 – -9.93	**<0.001**
timestep	4.642	4.013 – 5.270	**<0.001**	0.223	0.200 – 0.247	**<0.001**	3.453	3.015 – 3.892	**<0.001**
mother ID [2]	-0.082	-0.216 – 0.051	0.227	-0.012	-0.025 – 0.000	0.057	-0.164	-0.329 – 0.001	0.051
mother ID [3]	-0.012	-0.081 – 0.058	0.739	0.002	-0.005 – 0.008	0.655	-0.001	-0.088 – 0.085	0.979
tank position [middle]	0.027	-0.053 – 0.108	0.504	0.001	-0.007 – 0.008	0.887	0.032	-0.068 – 0.133	0.529
tank position [wall]	0.022	-0.063 – 0.107	0.614	-0.003	-0.011 – 0.005	0.465	-0.055	-0.161 – 0.052	0.314
tank system [2]	0.048	-0.043 – 0.139	0.298	0.004	-0.005 – 0.012	0.414	0.129	0.016 – 0.242	**0.026**
tank system [3]	0.029	-0.067 – 0.126	0.551	-0.000	-0.010 – 0.009	0.926	0.012	-0.108 – 0.132	0.842
tank system [4]	-0.001	-0.093 – 0.091	0.984	-0.004	-0.012 – 0.005	0.430	0.044	-0.070 – 0.158	0.452
**Random Effects**									
σ^2	0.04234			0.00005			0.02089		
τ_{00}	0.00758 id.2			0.00010 id.2			0.01681 id.2		
τ_{11}	0.37576 id.timestep			0.00180 id.timestep			0.15382 id.timestep		
	6.68184 id.I(timestep^2)			0.01845 id.I(timestep^2)			3.12505 id.I(timestep^2)		
ICC	0.19557			0.50830			0.16787		
N	45 id			45 id			45 id		
Observations	1239			1239			1239		
Marginal R^2 / Conditional R^2	0.151 / 0.317			0.399 / 0.705			0.281 / 0.402		

Table S7. Output for linear and quadratic models of behavioral entropy (calculated on a grid of 20 clusters obtained via UMAP dimension reduction and watershed segmentation) regressed against time (in days) as the continuous fixed predictor of interest. The quadratic model contains an additional term for $time^2$ as a continuous fixed predictor. Mother ID, tank position, and tank system are included as categorical fixed predictors, and fish ID is included as a random factor with varying intercepts and linear coefficients (as well as varying quadratic coefficients in the case of the quadratic model). P-values less than a 0.05 significance threshold are bolded.This table is related to the STAR Methods subsection '*Behavioral entropy measure*'.

	**Behavioral entropy (linear model – day interval)**			**Behavioral entropy (quadratic model – day interval)**		
Predictors	*Estimates*	*CI*	*p*	*Estimates*	*CI*	*p*
(Intercept)	1.79	1.65 – 1.92	**<0.001**	1.76	1.32 – 2.21	**<0.001**
timestep	0.87	0.21 – 1.53	**0.010**	7.16	5.45 – 8.88	**<0.001**
mother ID [2]	-0.10	-0.19 – -0.00	**0.049**	-1.08	-1.69 – -0.47	**0.001**
mother ID [3]	-0.07	-0.12 – -0.02	**0.006**	-0.26	-0.58 – 0.06	0.113
tank position [middle]	0.01	-0.05 – 0.06	0.819	-0.06	-0.43 – 0.32	0.771
tank position [wall]	0.02	-0.04 – 0.08	0.562	-0.24	-0.63 – 0.16	0.240
tank system [2]	0.06	-0.00 – 0.13	0.064	0.26	-0.16 – 0.68	0.225
tank system [3]	0.00	-0.07 – 0.07	0.912	-0.14	-0.58 – 0.31	0.549
tank system [4]	-0.03	-0.10 – 0.04	0.368	-0.15	-0.58 – 0.27	0.475
timestep^2				-21.67	-26.08 – -17.25	**<0.001**
**Random Effects**						
σ^2	0.07			0.05		
τ_{00}	0.16 $_{id}$			0.25 $_{id.2}$		
τ_{11}	4.63 $_{id.timestep}$			29.14 $_{id.timestep}$		
				170.19 $_{id.I(timestep\^2)}$		
ICC	0.35			0.94		
N	45 $_{id}$			45 $_{id}$		
Observations	1239			1239		
Marginal R^2 / Conditional R^2	0.061 / 0.385			0.128 / 0.948		

Table S8. Table of Akaike Information Criteria (AIC) and Bayesian Information Criteria (BIC) used to compare linear and quadratic models using day-length intervals (see Tables S5, S6, and S7) for each response. This table is related to the STAR Methods subsections '*Statistical models for basic behavioral measures*' and '*Behavioral entropy measure*'.

**Model**	**ΔAIC**	**ΔBIC**
Step length CoV quadratic (day interval)	0.00	0.00
Step length CoV linear (day interval)	163.05	157.93
Turning angle CoV quadratic (day interval)	0.00	0.00
Turning angle CoV linear (day interval)	149.24	144.12
Distance to tank wall CoV quadratic (day interval)	0.00	0.00
Distance to tank wall CoV linear (day interval)	201.18	196.06
Behavioral entropy quadratic (day interval)	0.00	0.00
Behavioral entropy linear (day interval)	172.00	166.87

Figure S2. Developmental arcs of behavioral plasticity calculated as either the coefficient of variation in a single behavioral metric (A-C) or as a measure of behavioral entropy across the entire movement repertoire (D) for the first 28 days of development. This figure relates to Fig 1 and Fig 3 in the text; in contrast to Fig 1 and Fig 3 in the main text, which calculates coefficients of variation or entropy over one-hour intervals, however, this figure corresponds to coefficients of variation or entropy that were calculated over one-day intervals. Note that, over these scales, the length of time over which data were aggregated has no bearing on the overall qualitative results (see also Tables S5-8).

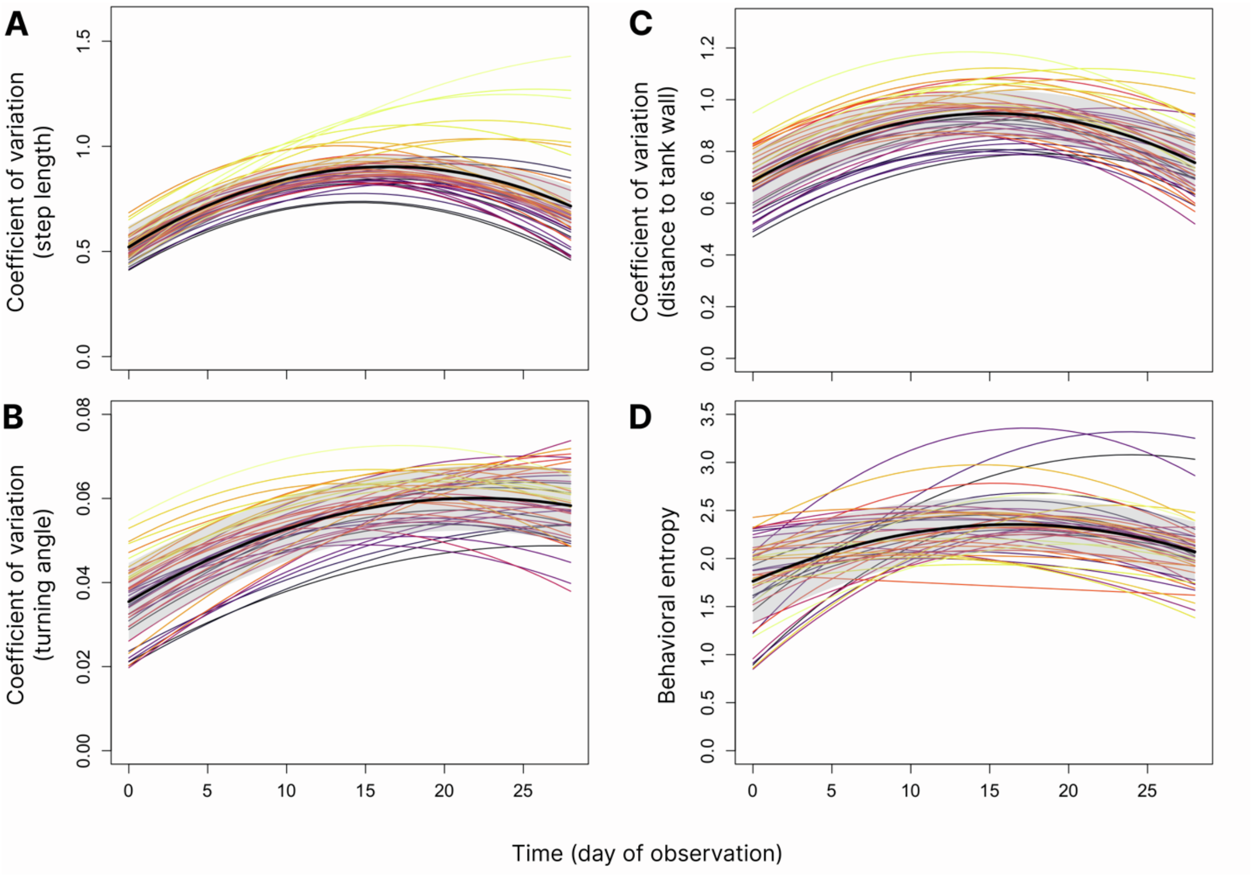

Behavioral entropy measures calculated across varying cluster numbers and with multiple clustering algorithms

Table S9. Output for linear models of behavioral entropy (calculated on a grid of 7 and 10 clusters obtained via UMAP dimension reduction and watershed segmentation) regressed against time (in hours) as the continuous fixed predictor of interest. Mother ID, tank position, and tank system are included as categorical fixed predictors, and fish ID is included as a random factor with varying intercepts and slopes. P-values below a 0.05 significance threshold are bolded. This table is related to the STAR Methods subsection '*Behavioral entropy measure*'.

	**Behavioral entropy – 7 clusters (linear model)**			**Behavioral entropy – 10 clusters (linear model)**		
Predictors	*Estimates*	*CI*	*p*	*Estimates*	*CI*	*p*
(Intercept)	0.61	0.53 – 0.69	**<0.001**	1.36	1.25 – 1.46	**<0.001**
timestep	0.03	-0.00 – 0.06	0.081	0.06	-0.00 – 0.12	0.070
mother ID [2]	-0.01	-0.12 – 0.09	0.800	-0.10	-0.19 – -0.01	**0.024**
mother ID [3]	-0.20	-0.26 – -0.15	**<0.001**	-0.12	-0.17 – -0.08	**<0.001**
tank position [middle]	-0.01	-0.07 – 0.05	0.724	-0.00	-0.06 – 0.05	0.954
tank position [wall]	0.01	-0.05 – 0.08	0.716	0.00	-0.05 – 0.06	0.873
tank system [2]	0.02	-0.05 – 0.09	0.661	0.06	0.00 – 0.13	**0.041**
tank system [3]	-0.01	-0.08 – 0.07	0.812	0.01	-0.06 – 0.07	0.844
tank system [4]	-0.01	-0.08 – 0.06	0.813	-0.02	-0.08 – 0.04	0.472
**Random Effects**						
σ^2	0.06			0.09		
τ_{00}	0.02 $_{id}$			0.09 $_{id}$		
τ_{11}	0.01 $_{id.timestep}$			0.05 $_{id.timestep}$		
ICC	0.17			0.23		
N	45 $_{id}$			45 $_{id}$		
Observations	9861			9861		
Marginal R^2 / Conditional R^2	0.129 / 0.280			0.051 / 0.273		

Table S10. Output for quadratic models of behavioral entropy (calculated on a grid of 7 and 10 clusters obtained via UMAP dimension reduction and watershed segmentation) regressed against time (in hours) as the continuous fixed predictor of interest, including a time^2 quadratic term. Mother ID, tank position, and tank system are included as categorical fixed predictors, and fish ID is included as a random factor with varying intercepts, linear, and quadratic coefficients. P-values less than a 0.05 significance threshold are bolded. This table is related to the STAR Methods subsection '*Behavioral entropy measure*'.

	**Behavioral entropy – 7 clusters (quadratic model)**			**Behavioral entropy – 10 clusters (quadratic model)**		
Predictors	*Estimates*	*CI*	*p*	*Estimates*	*CI*	*p*
(Intercept)	0.50	0.39 – 0.61	**<0.001**	1.38	1.02 – 1.74	**<0.001**
timestep^2	-0.11	-0.15 – -0.07	**<0.001**	-0.27	-0.34 – -0.20	**<0.001**
timestep	0.29	0.20 – 0.37	**<0.001**	0.66	0.47 – 0.86	**<0.001**
mother ID [2]	-0.17	-0.32 – -0.02	**0.030**	-0.87	-1.37 – -0.38	**0.001**
mother ID [3]	-0.10	-0.18 – -0.02	**0.014**	-0.23	-0.48 – 0.03	0.088
tank position [middle]	0.00	-0.09 – 0.10	0.919	-0.08	-0.39 – 0.22	0.580
tank position [wall]	0.03	-0.06 – 0.13	0.488	-0.24	-0.56 – 0.08	0.138
tank system [2]	-0.04	-0.14 – 0.06	0.460	0.21	-0.13 – 0.54	0.234
tank system [3]	-0.09	-0.20 – 0.02	0.115	-0.15	-0.51 – 0.21	0.413
tank system [4]	-0.03	-0.13 – 0.08	0.612	-0.15	-0.49 – 0.19	0.400
**Random Effects**						
σ^2	0.06			0.07		
τ_{00}	0.01 $_{id.2}$			0.17 $_{id.2}$		
τ_{11}	0.08 $_{id.timestep}$			0.44 $_{id.timestep}$		
	0.02 $_{id.I(timestep^{\wedge}2)}$			0.05 $_{id.I(timestep^{\wedge}2)}$		
ICC	0.70			0.91		
N	45 $_{id}$			45 $_{id}$		
Observations	9861			9861		
Marginal R^2 / Conditional R^2	0.037 / 0.708			0.094 / 0.920		

Table S11. Output for linear and quadratic models of behavioral entropy (calculated on a grid of 20 clusters obtained via k-means clustering) regressed against time (in hours) as the continuous fixed predictor of interest. The quadratic model contains an additional quadratic term for time^2 as a continuous fixed predictor. Mother ID, tank position, and tank system are included as categorical fixed predictors, and fish ID is included as a random factor with varying intercepts and linear coefficients (as well as varying quadratic coefficients in the case of the quadratic model). P-values less than a 0.05 significance threshold are bolded. This table is related the STAR Methods subsection '*Behavioral entropy measure*'.

	**Behavioral entropy calculated with k-means (linear model)**			**Behavioral entropy calculated with k-means (quadratic model)**		
Predictors	*Estimates*	*CI*	*p*	*Estimates*	*CI*	*p*
(Intercept)	1.91	1.79 – 2.04	**<0.001**	1.94	1.52 – 2.37	**<0.001**
timestep	0.07	0.00 – 0.15	**0.038**	0.80	0.57 – 1.02	**<0.001**
mother ID [2]	-0.08	-0.17 – 0.02	0.114	-1.07	-1.66 – -0.49	**<0.001**
mother ID [3]	-0.13	-0.18 – -0.08	**<0.001**	-0.25	-0.56 – 0.06	0.115
tank position [middle]	0.01	-0.05 – 0.07	0.684	-0.11	-0.47 – 0.25	0.543
tank position [wall]	0.02	-0.04 – 0.08	0.488	-0.27	-0.65 – 0.11	0.166
tank system [2]	0.04	-0.02 – 0.11	0.200	0.24	-0.16 – 0.65	0.243
tank system [3]	0.01	-0.06 – 0.08	0.721	-0.18	-0.61 – 0.24	0.401
tank system [4]	-0.02	-0.08 – 0.05	0.659	-0.16	-0.57 – 0.24	0.432
timestep^2				-0.32	-0.40 – -0.24	**<0.001**
**Random Effects**						
σ^2	0.11			0.09		
τ_{00}	0.13 $_{id}$			0.24 $_{id.2}$		
τ_{11}	0.06 $_{id.timestep}$			0.59 $_{id.timestep}$		
				0.07 $_{id.I(timestep^2)}$		
ICC	0.25			0.92		
N	45 $_{id}$			45 $_{id}$		
Observations	9861			9861		
Marginal R^2 / Conditional R^2	0.046 / 0.285			0.099 / 0.927		

Figure S3. Developmental arcs of plasticity in movement repertoires of Amazon mollies over the first 28 days of life, quantified as behavioral entropy. Shown here are graphs of behavioral entropy through time, where entropy is calculated using a range of cluster numbers and over two alternative clustering algorithms. Note that the general pattern of entropy remains unchanged across cluster numbers and specific clustering algorithms: (A) entropy calculated across only 7 clusters, obtained via UMAP dimension reduction and watershed segmentation, (B) entropy calculated across 10 clusters, obtained via UMAP dimension reduction and watershed segmentation, (C) entropy calculated across 20 clusters, obtained via UMAP dimension reduction and watershed segmentation (as in Fig 3 main text), and (D) entropy calculated across 20 clusters, obtained via k-means clustering. This figure is related to Fig 3 in the main text.

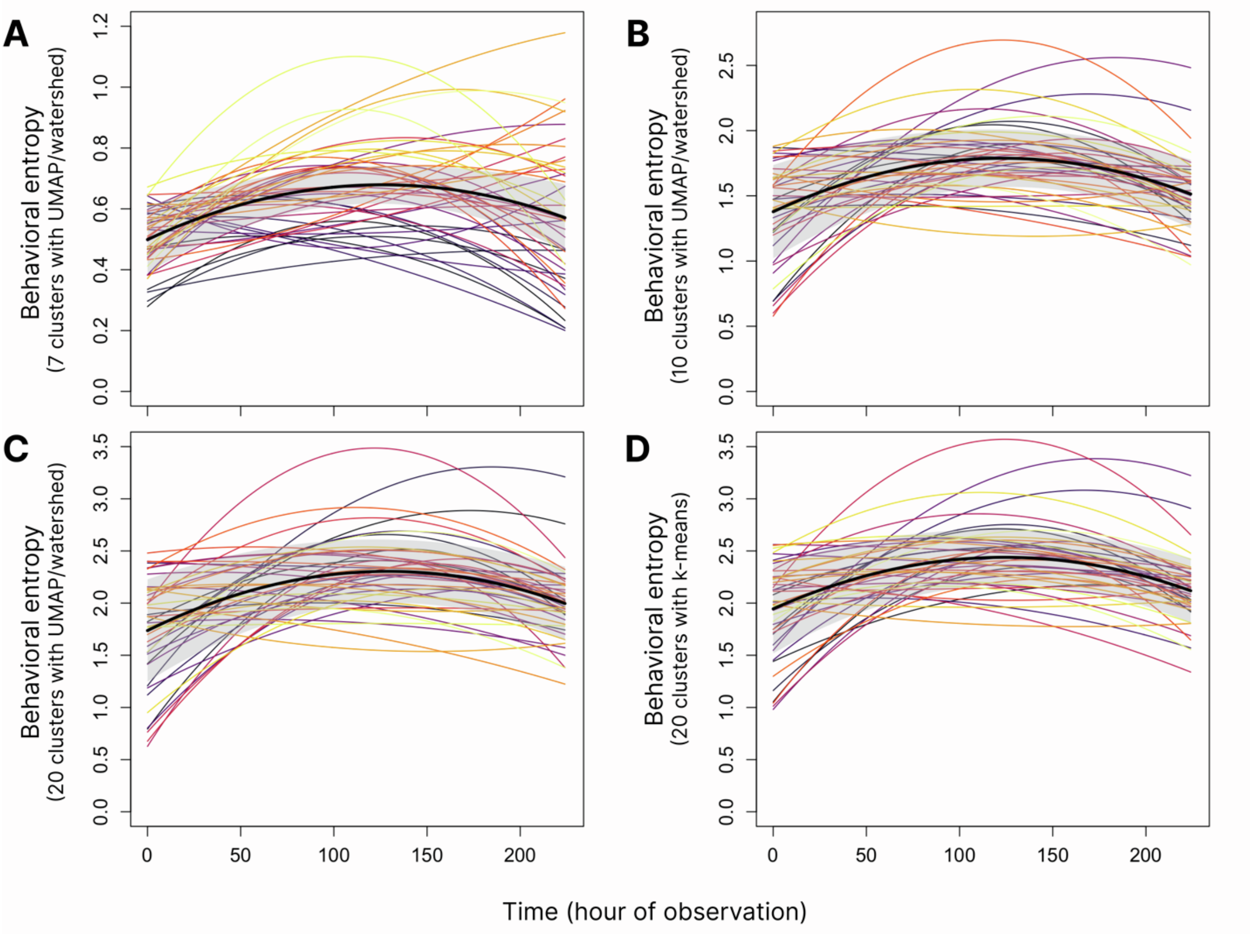

Movie of simultaneous movement in physical space and behavioral phenotype space for low- and high-entropy individuals

Video S1. A 5-minute movie in which three low entropy individuals (A) and three high entropy individuals (B) are shown exhibiting a continuous track of behavior over a 30-minute real-time sequence (6x speed) in both physical tank space (triangular shapes on the left of each panel) and clustered behavioral phenotype (UMAP) space. This movie is related to and referenced in the '*Behavioral plasticity as entropy in multi-dimensional behavioral phenotype space*' section in the main text.

Supplemental references

1. Scherer, U., Ehlman, S.M., Bierbach, D., Krause, J., and Wolf, M. (2023). Reproductive individuality of clonal fish raised in near-identical environments and its link to early-life behavioral individuality. *Nature Communications 14*, 7652. https://doi.org/10.1038/s41467-023-43069-6. (*referenced in-text as reference 27*)
